# Spatiotemporal Modeling of Mangrove Carbon Stock Along Pakistan’s Coast Using Multi-Sensor Sentinel and Landsat Data

**DOI:** 10.3390/s26134117

**Published:** 2026-06-29

**Authors:** Junaid Ahmad Qadri, Asif Sajjad, Aqib Hassan Ali Khan

**Affiliations:** 1 Department of Environmental Sciences, Faculty of Biological Sciences, Quaid-i-Azam University, Islamabad 45320, Pakistan; 2 International Research Center in Critical Raw Materials and Advanced Industrial Technologies, Universidad de Burgos, 09001 Burgos, Spain

**Keywords:** coastal ecosystem monitoring, biomass estimation, environmental stress, remote sensing, Google Earth Engine, light use efficiency model

## Abstract

**Highlights:**

**What are the main findings?**

- Pakistan’s arid-zone mangroves maintain an average carbon stock of 31.95 Mg C ha^−1^ and exhibit “pulse resilience,” where a drought-induced 11.11% decline in 2021 was followed by rapid biomass recovery during the 2022 flood pulse.
- Vapor pressure deficit (VPD), rather than temperature variation, serves as the decisive meteorological driver of carbon sequestration, showing a significant inverse relationship (R^2^ = 0.76) between atmospheric moisture demand and stock density.

**What are the implications of the main findings?**

- The integration of process-based light use efficiency (LUE) modeling establishes a high-precision baseline for national blue carbon accounting and REDD+ initiatives within hyper-arid, data-scarce coastal environments.
- A multi-sensor fusion framework provides a robust, cost-neutral methodology for tracking the functional integrity and carbon sink stability of specialized halophytic ecosystems amidst escalating climatic variability.

**Abstract:**

This study quantifies coastal mangrove carbon stocks and their interannual variability along the Pakistan coastline by developing a multi-sensor fusion framework integrated with a process-based light use efficiency (LUE) modeling approach. To ensure high-cadence monitoring and overcome persistent cloud cover over the Indus Delta, data from multiple satellite sensors including Landsat 8/9 and Sentinel-2 within Google Earth Engine were utilized. Sentinel-2-derived Normalized Difference Vegetation Index (NDVI) data composited for the January–March period was processed to estimate vegetation productivity. Field-based validation of biomass estimates was conducted using 57 georeferenced sampling points, cross-compared with Sentinel-2 data. Mangrove extent was delineated through land use and land cover (LULC) classification into water bodies, mangroves, mudflats, land parcels, and sand surfaces. The LUE model incorporated environmental stress scalars, including temperature, vapor pressure deficit (VPD), salinity, and photosynthetically active radiation (PAR) to estimate gross primary productivity and derive total biomass, which was subsequently converted into carbon stocks. Results indicate a mean carbon stock of 31.95 Mg C ha^−1^ (equivalent to 117.3 Mg CO_2_ ha^−1^), with significant interannual variation (coefficient of variation = 19.8%). A significant decline in carbon stocks was observed in 2021 (−11.11%; 3.56 Mg C ha^−1^), corresponding to a reduction in NDVI value (0.55 compared to 0.58 in other years). Spatial analysis revealed substantial heterogeneity in carbon distribution (20.51 to 55.93 Mg C ha^−1^), primarily influenced by localized salinity gradients and water stress conditions. This study mapped mangrove extent, quantified environmental stress, and estimated carbon stocks across Pakistan’s coast from 2020 to 2024, delivering a spatially resolved, multi-year baseline for coastal carbon assessment and ecosystem monitoring in arid tidal environments.

## 1. Introduction

Mangrove forests represent some of the most physiologically intricate and productive biomes globally, functioning as critical biogenic buffers for tropical and subtropical coastal zones [1]. These unique halophytic ecosystems thrive within the dynamic ecotone between terrestrial and marine environments, persisting under extreme conditions characterized by hypersalinity, waterlogging, and anaerobic soils in intertidal regions [2,3]. Mangroves cover less than 1% of global forests; however, acting as a major carbon sink, these ecosystems can sequester carbon at rates 3 to 4 times higher than most land-based tropical and temperate forests [4,5,6]. As structurally specialized and functionally diverse ecosystems, mangroves act as primary ecosystem engineers, driving biophysical landscape modification and facilitating nutrient exchange across the land–sea interface [7,8,9,10].

This ecological significance is underpinned by their remarkable capacity to adapt to extreme coastal environments. Mangrove ecosystems are also known for their ability to persist under periodic tidal inundation, anoxic sediments, and high sedimentation rates [11,12,13]. These ecosystems exhibit a suite of specialized physiological and morphological adaptations, reflecting both functional and structural evolutionary strategies. Such adaptations include ion exclusion and salt secretion mechanisms, specialized aerial root systems (including pneumatophores and prop roots), and viviparous propagules, enabling them to establish and thrive in environments largely inhospitable to most terrestrial plant species [7,14,15].

Beyond their adaptive resilience and ecological complexity, mangrove forests provide extensive ecosystem services crucial for coastal sustainability. Their complex subterranean and aerial root networks act as natural bio-shields, diffusing wave energy to protect shorelines from erosion and extreme meteorological events such as storm surges, cyclones, and tsunamis [16,17]. Mangrove ecosystems support high levels of biodiversity by functioning as nursery grounds for fish, crustaceans, and mollusks, while simultaneously providing habitat for birds, reptiles, and mammals [18,19,20]. Through these functions’ roles, mangroves act as keystone ecosystems for coastal food webs and fisheries productivity [21]. Their ecological and socio-economic significance aligns directly with global sustainability frameworks, particularly Sustainable Development Goal 14, which emphasizes the conservation and sustainable use of marine and coastal ecosystems [16,22].

On the global scale, mangroves extend across 152,000 km^2^ of coastline, predominantly distributed between 25° N and 25° S, where they form a critical component of tropical and subtropical coastal landscapes [23,24,25]. Despite their ecological and socio-economic significance, these ecosystems have experienced profound degradation in recent decades, primarily driven by anthropogenic pressures such as coastal urbanization, port and harbor development, the conversation of mangrove habitats for aquaculture [26], fuelwood extraction, and hydrological alterations bridged with river regulation and irrigation schemes [27,28].

In addition to these human-induced stressors, climate change further exacerbates mangrove vulnerability through sea-level rise, increasing sea surface temperatures, and changes in precipitation patterns. These impacts are mainly within arid and semi-arid domains where riverine or freshwater inputs are already constrained [29,30]. Although recent studies indicate a deceleration and, in some regions, stabilization of global mangrove attrition rates, largely attributable to improved conservation frameworks and active restoration initiatives, significant regional disparities persist [31,32]. This variability highlights the critical need for spatially explicit, high-resolution, and temporally consistent monitoring frameworks to better understand mangrove dynamics, quantify carbon stocks, and support evidence-based conservation and management strategies.

Building upon these global patterns, Asia supports approximately 1.19 million hectares of mangrove forests, accounting for nearly 7% of the global extent, with predominant distribution along the coastlines of South Asia including India, Bangladesh, Pakistan, and Sri Lanka [16,33,34]. Within this regional context, Pakistan’s coastline, stretching over 1000 km, harbors one of the largest arid-climate mangrove systems in the world, primarily concentrated in the Indus Delta. These ecosystems, dominated by species such as *Avicennia marina*, function as critical socio-ecological lifelines for the coastal communities of Sindh and Balochistan, supporting livelihoods, fisheries, and coastal protection [35,36].

In contrast to widespread global mangrove decline, Pakistan has demonstrated a notable expansion in mangrove cover over recent decades, largely driven by policy interventions and large-scale restoration initiatives [37]. However, despite these gains, mangrove ecosystems in Pakistan remain vulnerable to both anthropogenic pressures and climate-induced stressors, necessitating continuous, spatially explicit monitoring to assess their long-term sustainability and functional dynamics [34].

Accurate assessment of mangrove carbon stocks requires understanding the distribution of carbon between above ground and below ground biomass pools, particularly in arid coastal environments where salinity and water stress strongly influence biomass allocation and carbon sequestration [9,35,36]. Recent studies have shown that integrating satellite observations with advanced modeling approaches can substantially improve biomass and carbon stock estimation accuracy [37,38]. However, research along Pakistan’s coastline has primarily focused on mangrove extent and land-cover dynamics, while spatially explicit assessments of mangrove carbon stocks remain limited [9,34]. Addressing this gap is essential for developing reliable carbon inventories and supporting national climate mitigation initiatives. Therefore, this study provides a calibrated baseline assessment of mangrove carbon stocks by integrating environmental stress factors with a process-based LUE framework following IPCC wetland guidelines.

In this context, remote sensing has emerged as an integral component for monitoring mangrove extent and physiological vigor across multi-scalar dimensions, mitigating the logistical constraints of field-based assessments in intertidal environments [39,40]. Multispectral platforms, specifically Landsat and Sentinel, facilitate long-term, spatially consistent observations offering a robust and standardized analytical framework to delineate spatiotemporal fluctuations in mangrove extent, spectral vegetation indices, and canopy structural dynamics [41,42]. Furthermore, advances in cloud-computing and machine learning algorithms have revolutionized accuracy of mangrove classification and change detection, allowing the integration of large satellite archives with robust statistical learning techniques such as Random Forests and support vector machines [43,44].

Beyond structural mapping, the integration of process-based light use efficiency (LUE) models provides a robust framework for quantifying ecosystem function, particularly for estimating aboveground biomass and carbon dynamics [45]. By establishing a mechanistic relationship between photosynthetically active radiation (PAR) and vegetation indices, LUE models enable improved assessment of carbon sequestration processes in mangrove ecosystems [46,47]. Despite these advancements, there remains a critical gap in high-resolution, spatiotemporally explicit assessments of mangrove carbon dynamics along the Pakistan coastline, particularly under arid environmental conditions. Addressing this gap is essential for improving national carbon inventories, supporting REDD+ initiatives, and informing adaptive coastal management strategies in the face of climate change.

Therefore, the objectives of this study were to (1) map the spatiotemporal distribution of mangrove forests along Pakistan’s coast using multi-sensor Sentinel and Landsat imagery; (2) quantify key environmental stress factors, including temperature, water availability, salinity, and photosynthetically active radiation (PAR), affecting mangrove photosynthetic efficiency; (3) estimate aboveground biomass and carbon stocks through an integrated light use efficiency (LUE) modeling framework incorporating site-specific stress scalars; and (4) generate a spatiotemporal baseline of mangrove carbon stocks for Pakistan’s coastal mangrove ecosystems.

## 2. Materials and Methods

### 2.1. Study Area

**Figure 1:**
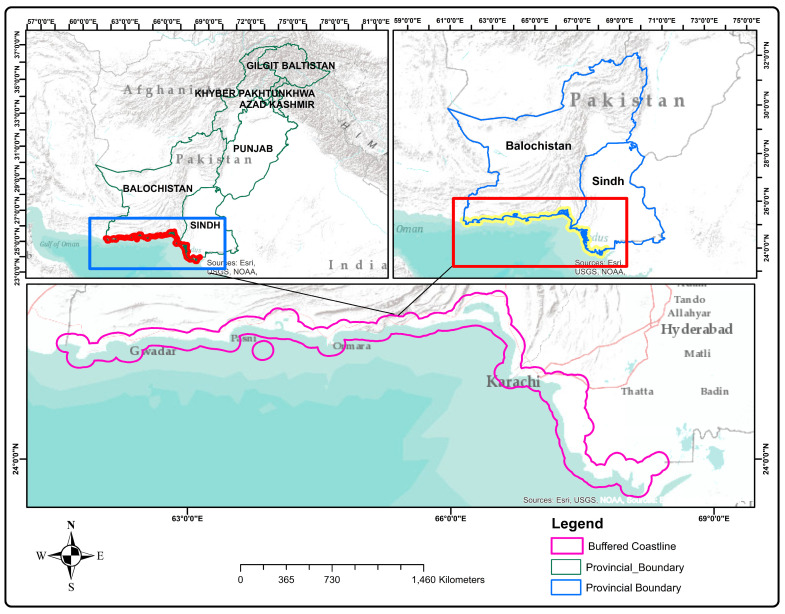
Geographical location of the study area along the Pakistan coast.

The study area encompasses approximately 1050 km of Pakistan’s coastline, extending from Iranian border in the west to the Indian border in the east. This coastal zone comprises two distinct hydrographic and geomorphological regions: the Balochistan Coast and the Sindh Coast (Figure 1). The Balochistan coast, spanning nearly 700 km, is characterized by rocky cliffs and headland embayment’s. The mangrove forests in the region are spatially confined and occur in discrete pockets, primarily within sheltered environments such as Miani Hor, Kalmat Khor, and Gwadar Bay. In contrast, the Sindh coast, extending approximately 350 km, is dominated by the Indus Delta. This region is characterized by an extensive network of tidal creeks, mudflats, and intertidal zones, which support the largest arid-climate mangrove forests in the region. These forests are predominantly composed of *Avicennia marina*, reflecting adaptation to high salinity and limited freshwater inflows.

### 2.2. Data

This study used multiple remote sensing datasets to assess mangrove dynamics along the Sindh and Balochistan coasts during the 2020–2024 period. Landsat 8 and 9 imageries acquired from the United States Geological Survey (USGS, Reston, VA, USA) were utilized for long-term coastal mapping and LULC classification [48,49]. LULC classification was performed using harmonized Landsat 8/9 (OLI/OLI-2) Collection 2 Level 2 data. In parallel, Sentinel-2 Multispectral Instrument (MSI) Level-2A multispectral data and Sentinel-1 C-band Synthetic Aperture Radar (SAR) data (10 m spatial resolution), provided by the European Space Agency (ESA, Paris, France), were used for high-resolution mangrove delineation; NDVI computation; and estimates of aboveground biomass (AGB), belowground biomass (BGB), total aboveground biomass (TAB), and carbon stocks [50]. To ensure consistency and minimize environmental noise, image acquisition was restricted to the dry pre-monsoon period (January–March), thereby reducing the effects of cloud cover, atmospheric aerosol loading, and tidal inundation variability. This temporal window is particularly suitable for arid and semi-arid mangrove systems, where dry-season vegetation indices show strong correspondence with annual biomass due to salinity-driven growth constraints [7,51].

### 2.3. Methodology

**Figure 2:**
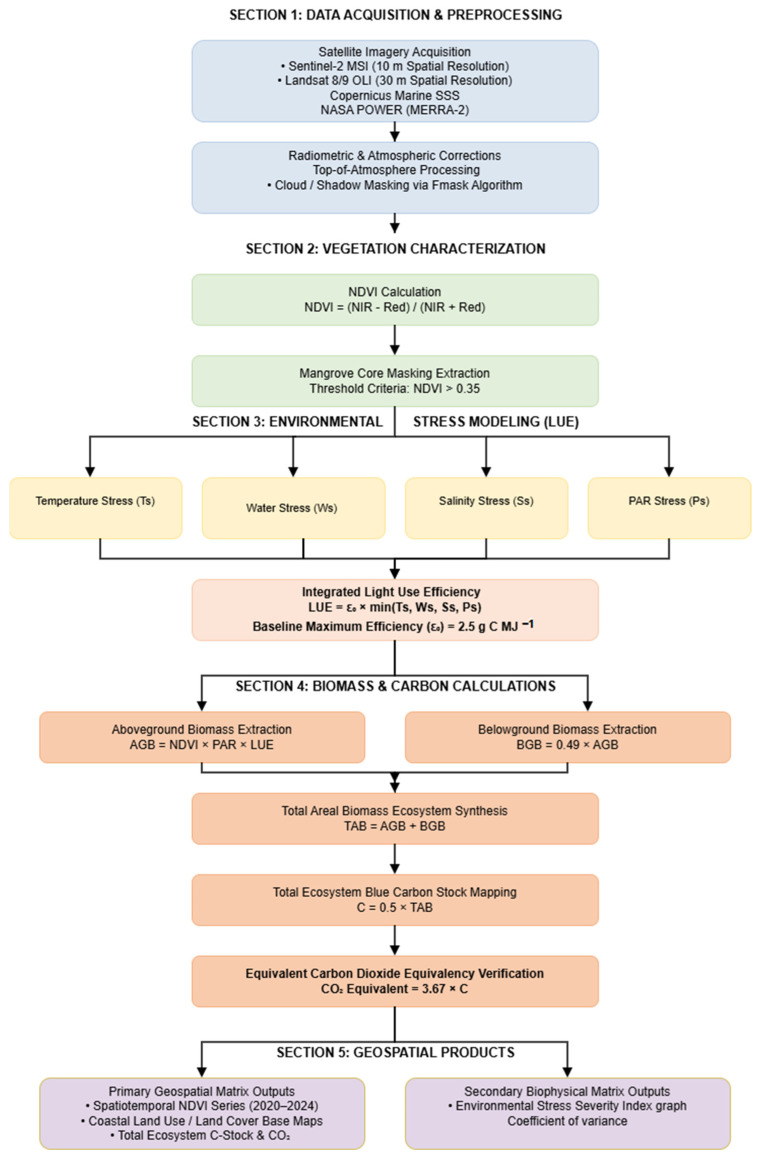
Methodological workflow for mangrove delineation, biomass estimation, and carbon stock assessment.

The overall methodological workflow adopted for mangrove delineation, biomass estimation, and carbon stock assessment is presented in Figure 2, which integrates remote sensing, machine learning, and productivity-based modeling approaches.

#### 2.3.1. NDVI-Based Mangrove Delineation

Mangrove extent and vegetation condition were delineated using the Normalized Difference Vegetation Index (NDVI) derived from Sentinel-2 imagery provided by the European Space Agency (ESA, Paris, France). NDVI serves as a robust spectral indicator of vegetation vigor by exploiting the contrast between high near-infrared (NIR) reflectance and red-band absorption associated with chlorophyll activity. The index was calculated in Equation (1) as

(1) $$NDVI=\frac{NIR-Red}{NIR+Red}$$

where NIR and Red represent reflectance values in the near-infrared and red spectral bands, respectively. NDVI values range from −1 to +1, with higher values indicating denser and healthier vegetation.

A uniform NDVI threshold of 0.35 was applied to delineate mangrove canopies from non-vegetated and sparsely vegetated surfaces under arid coastal conditions. Rather than applying a single static mangrove mask throughout the study period, the thresholding procedure was independently implemented for each annual January–March composite to capture interannual variability in canopy extent. The threshold value was selected based on published regional studies of mangrove vegetation indices and further verified through iterative visual comparison with high-resolution imagery available in Google Earth Pro 7.3 (Google LLC, Mountain View, CA, USA) [52].

#### 2.3.2. LULC Classification Using Random Forest

Mangrove extent and coastal land cover dynamics were further quantified using a Random Forest (RF) classifier applied to Landsat data (2020–2024). RF is an ensemble-based machine learning algorithm known for its robustness against noise, its ability to handle high-dimensional spectral data, and its robustness against overfitting in heterogeneous coastal environments. RF leverages a consensus-voting mechanism across multiple dimension trees to enhance thematic accuracy. Five main LULC classes were identified: mangrove, water, mudflats, land parcels, and sand. To ensure spatiotemporal comparability and accurately track mangrove expansion or degradation, these class definitions were maintained consistently across the 2020–2024 study period.

Training samples for all LULC classes were generated from pre-processed Landsat mosaics using a stratified random sampling approach to ensure representative spatial coverage across the study area. The sampled points were validated through expert visual interpretation and cross-checked with high-resolution Google Earth imagery to minimize spectral confusion among mangroves, water, mudflats, land parcels, and sand surfaces. Approximately 1000 samples were used for dominant classes (mangrove, water, and mudflats), while fewer samples were assigned to less extensive classes (land parcels and sand) due to their limited spatial extent. The Random Forest classifier was implemented in Google Earth Engine (Google LLC, Mountain View, CA, USA) using 650 decision trees, and final class labels were determined using a majority-vote ensemble rule across all trees, ensuring robust and stable classification outputs for subsequent analysis.

RF The classification logic is expressed as

(2) $$\hat{y}=mode\left\{h_{1}\left(x\right),h_{2}\left(x\right)\ldots \ldots h_{T}\left(x\right)\right\}$$

where $\hat{y}$ is the predicted class, $h_{T}(x)$ is the prediction of the t-th decision tree, and T is the total number of trees.

The resulting LULC mangrove extent maps provided a spatial framework for integrating NDVI-based productivity modeling and carbon stock estimation.

#### 2.3.3. Biomass and Carbon Estimation

Mangrove biomass and carbon stocks were estimated to use a productivity-based light use efficiency (LUE) framework that links vegetation productivity to photosynthetically active radiation (PAR) and environmental stress constraints [53]. AGB was calculated in Equation (3) as

(3) AGB=NDVI×PAR×LUE

where PAR is photosynthetically active radiation (MJ m^−2^ day^−1^), fPAR is the fraction of absorbed photosynthetically active radiation, and LUE is light use efficiency (g DM MJ^−1^). The fraction of absorbed photosynthetically active radiation (fPAR) was derived from NDVI using established empirical relationships between vegetation indices and canopy radiation absorption [46,54].

The biomass estimation framework was adapted from established light use efficiency (LUE) approaches that relate vegetation productivity to absorbed photosynthetically active radiation and environmental constraints. Similar formulations have been widely applied in regional biomass and carbon assessments [46,53]. These studies provide a robust theoretical basis for translating remotely sensed vegetation characteristics into biomass estimates in data-scarce environments.

#### 2.3.4. LUE Formulation

The LUE was computed using Equation (4):

(4) $$LUE=\varepsilon_{0}\times T_{s}\times W_{s}\times S_{s}\times P_{s}$$

where $\varepsilon_{0}$ is the maximum light use efficiency (2.5 g DM MJ^−1^) [46], and $T_{s}$, $W_{s}$, $S_{s}$, and $P_{s}$ represent temperature stress, water stress, salinity stress, and radiation stress scalars, respectively, each ranging from 0 to 1.

The multiplicative formulation was adopted to represent the combined influence of environmental constraints on photosynthetic efficiency. This approach ensures that reductions in any individual environmental factor proportionally limit overall productivity. Furthermore, the structure follows the ecological principle that productivity is constrained by the most limiting environmental factor and has been widely applied in ecosystem productivity and carbon modeling studies.

Meteorological variables used in the LUE model were obtained from the NASA POWER database developed by the National Aeronautics and Space Administration (NASA, Washington, DC, USA). Air temperature and dew-point temperature derived from the MERRA-2 reanalysis product were used to calculate temperature and vapor pressure deficit stress functions, while photosynthetically active radiation (PAR) data were obtained from the CERES SYN1deg product.

#### 2.3.5. Environmental Stress Scalars

##### Temperature Stress ($T_{s}$)

Temperature thresholds (Tmin, Topt, and Tmax) were selected based on published physiological studies of mangrove species, particularly *Avicennia marina*, which dominates the mangrove ecosystems of Pakistan’s coastline [54,55,56]. Tmin (20 °C) represents the lower temperature limit below which photosynthetic activity and carbon assimilation begin to decline. Topt (28 °C) corresponds to the optimum temperature for maximum photosynthetic efficiency, while Tmax (35 °C) represents the upper thermal threshold beyond which heat stress and stomatal closure substantially reduce productivity.

Air temperature data were obtained from the NASA/POWER database and used to calculate the temperature stress scalar (Ts). Following established light use efficiency (LUE) modeling approaches adapted for mangrove ecosystems [55,56,57], Ts was calculated using Equation (5):

(5) $$T_{s}=\frac{\left(T-T_{min})(T-T_{max}\right)}{(T-T_{min})(T-T_{max})-(T-T_{opt}{)}^{2}}$$

##### Water Stress ($W_{s}$) via VPD

VPD thresholds were selected following established eco-physiological studies, representing optimal and stress-inducing atmospheric moisture conditions for mangrove productivity [53,58]. 

Following the approach of Zhou et al. [59], $W_{s}$ was calculated using Equation (6):

(6) $$VPD=e_{s}-e_{a}$$

The saturation vapor pressure ($e_{s}$) was calculated using the Tetens equation (Equation (7)), a widely accepted empirical relationship for estimating es as a function of temperature:

(7) $$e_{s}\left(T\right)=0.6108\times \exp\left(\frac{17.27\cdot T}{T+237.3}\right)$$

Actual vapor pressure $e_{a}$ was calculated using dew point temperature Equation (8):

(8) $$e_{a}\left(T_{dew}\right)=0.6108\times \exp\left(\frac{17.27\cdot T_{dew}}{T_{dew}+237.3}\right)$$

Atmospheric water stress (W_s) is captured via land surface temperature and vapor pressure deficit metrics, reflecting atmospheric moisture demands on the canopy.

##### Salinity Stress ($S_{s}$)

The salinity response coefficient (0.0047) was adopted from empirical studies on *Avicennia marina*, reflecting species-specific sensitivity to increasing salinity levels. Following the methodology of Lovelock et al. [60], the salinity stress factor was calculated using Equation (9) as

(9) $$S_{s}=1-\left(0.0047\times Salinity\right)$$

Daily mean Sea Surface Salinity (SSS) data for the 2020–2024 timeframe were obtained from the Copernicus Marine Environment Monitoring Service (CMEMS, Mercator Ocean International, Toulouse, France). This dataset provides continuous, gap-free Level 4 salinity fields at a spatial resolution of 1/8 degree (approximately 14 km). The product is generated by the Consiglio Nazionale delle Ricerche (CNR) through a multivariate optimal interpolation framework that blends surface observations from the SMOS and SMAP satellite missions with distributed in situ profile measurements. To minimize short-term atmospheric noise and capture distinct seasonal environmental trends, the daily SSS fields were aggregated into seasonal means, geographically clipped to the study area, and maintained in the WGS84 geographic coordinate system (EPSG:4326) for subsequent input into Formula (7).

The LUE framework accounts for both atmospheric and substrate-level water stress. Atmospheric water stress is represented through vapor pressure deficit (VPD), while substrate water limitation is represented through the salinity stress scalar. In intertidal mangrove environments, pore-water salinity is strongly coupled with coastal water salinity through regular tidal exchange, making Sea Surface Salinity a suitable proxy for root-zone osmotic stress.

##### PAR Stress ($P_{s}$)

A lower threshold constraint (0.3) was imposed to account for baseline metabolic activity under suboptimal radiation conditions. Following the methodology of Barr et al. [53], light saturation response, $P_{s}$, was calculated as in Equation (10):

(10) $$P_{s}=1-(0.0101\times PAR)$$

All stress scalars are constrained to [0, 1] to ensure physical validity; for the observed environmental ranges in this study, Ss and Ps operate well within these bounds.

#### 2.3.6. Biomass Estimation

AGB was modeled using the NDVI, PAR, and LUE parameters. This formulation represents a simplified productivity-based proxy for biomass estimation, adapted for data-scarce coastal environments where extensive field-based calibration is “limited” [46]. AGB was estimated using Equation (11):

(11) AGB=NDVI×PAR×LUE

The foundational architecture of Equation (9) relies on light use efficiency (LUE) frameworks, where variables such as the NDVI, photosynthetically active radiation (PAR), and maximum LUE function as primary drivers for regional aboveground biomass (AGB) estimation. This specific empirical formulation was adapted from established remote sensing protocols optimized for vegetation modeling [47]. These sources were selected because they provide robust scalable baselines for translating canopy reflectance into total accumulated biomass.

A root-to-shoot ratio of 0.49 was adopted for estimating belowground biomass. This value falls within the range reported for *Avicennia marina* growing under saline and arid environmental conditions and reflects the tendency of mangroves to allocate a larger proportion of biomass below ground in response to osmotic stress and limited freshwater availability [35,36,49].

BGB was calculated, using Equation (12), through the allometric relationship proposed by Cairns et al. [61], widely applied in mangrove studies, including the Sundarbans:

(12) BGB=0.49×AGB

The root-to-shoot ratio applied in this study is consistent with established allometric relationships for mangrove ecosystems. Total biomass (TAB) was calculated as in Equation (13):

(13) TAB=AGB+BGB

#### 2.3.7. Carbon Stock and CO_2_ Sequestration

The carbon fraction value of 0.5 was applied consistently with IPCC guidelines for woody biomass and has been widely applied in mangrove carbon stock assessments. Carbon stock was estimated using the following carbon fraction (Equation (14)):

(14) C=0.5×TAB

To evaluate climate mitigation potential and enable comparison with global greenhouse gas inventories, total carbon stocks were converted to CO_2_ equivalent using the molecular weight ratio of CO_2_ to carbon (44/12 = 3.67), as in Equation (15) [62]:

(15) $$CO_{2}=3.67\times C$$

All datasets (including Sentinel-2, Landsat, NASA/POWER, and Copernicus products) were spatially resampled, projected, and harmonized to a common coordinate system to ensure consistency and minimize spatial discrepancies during multi-source data integration within the LUE modeling framework.

#### 2.3.8. Ground Validation and Accuracy Assessment

**Figure 3:**
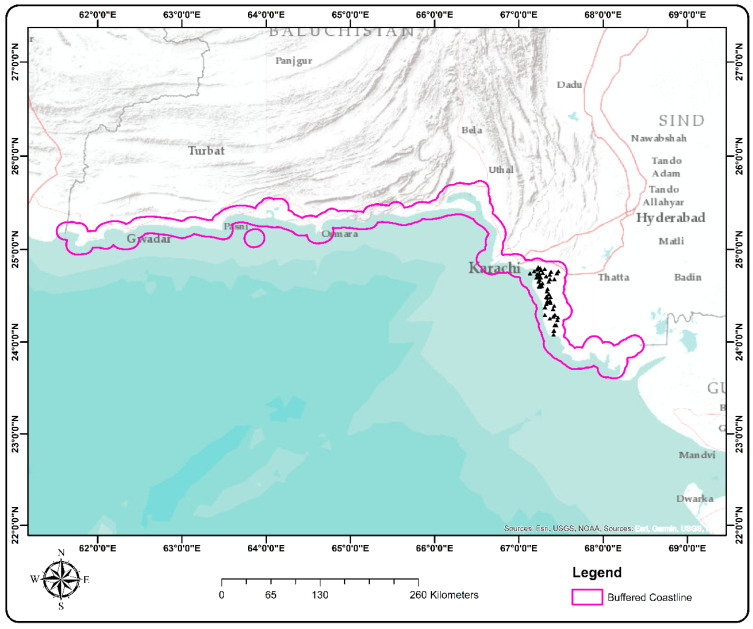
Spatial distribution of 57 ground points across study areas.

Ground reference points were distributed across major mangrove zones along the Sindh and Balochistan coast to capture spatial heterogeneity and ensure representative validation of classification outputs (Figure 3). A total of 57 ground reference validation points were used for accuracy assessment over the 2020–2024 study period (Table 1). These points were obtained from the National Institute of Oceanography (NIO), Karachi, and represent key mangrove environments, including information on geographic location and dominant vegetation characteristics. High-resolution Google Earth imagery and historical satellite data were used to verify site stability and ensure temporal consistency across the study period.

A stratified random sampling approach was applied to ensure balanced representation of all land cover classes and to minimize sampling bias. This ensured a reliable and statistically robust validation dataset for accuracy assessment.

Classification performance was evaluated using confusion matrix-based metrics, including overall accuracy (OA), producer’s accuracy (PA), user’s accuracy (UA), and the Kappa coefficient (κ).

The overall accuracy (OA) was calculated using Equation (16):

(16) $$OA=\frac{\sum x_{ii}}{n}$$

The Kappa coefficient (κ) was computed using Equation (17):

(17) $$K=\frac{n\sum x_{ii}-\sum (x_{i+}\cdot x_{+i})}{n^{2}-\sum (x_{i+}\cdot x_{+i})}\;$$

where $x_{ii}$ represents correctly classified samples (diagonal elements of the confusion matrix), $x_{i+}$ and $x_{+i}$ represent the marginal totals of rows and columns, and n is the total number of validation samples.

This multi-metric evaluation framework provides a robust assessment of classification accuracy and ensures reliable outputs for subsequent biomass estimation and carbon stock analysis.

##### Uncertainty and Model Robustness

The absence of extensive field-based biomass measurements introduces uncertainty in model calibration and represents a limitation of this study. However, the integration of multi-source satellite data and process-based modeling provides a robust framework for regional-scale assessment of mangrove dynamics. To evaluate model robustness, a sensitivity analysis of key model parameters was conducted under varying environmental conditions. This analysis was used to assess the stability of biomass and carbon stock estimates across the 2020–2024 study period.

## 3. Results

### 3.1. Spatiotemporal Distribution of Mangrove Extent and LULC Dynamics

**Figure 4:**
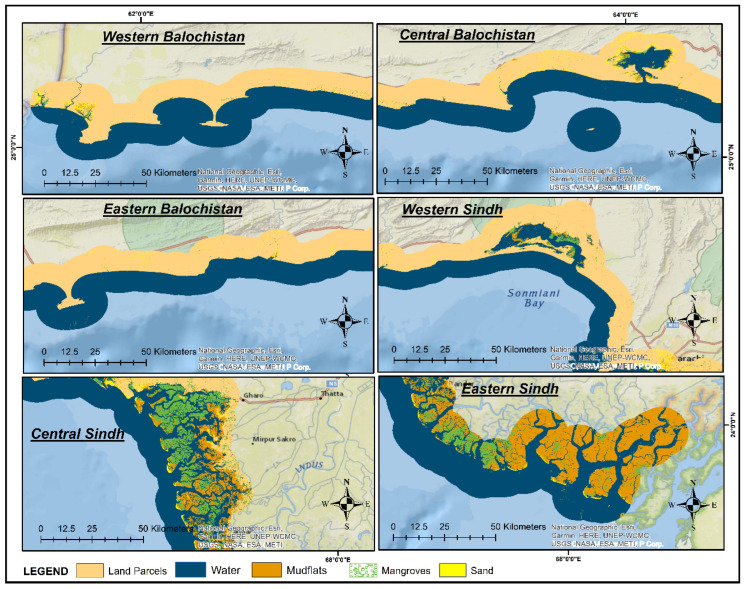
Spatial heterogeneity of mangrove distribution across distinct coastal zones of Pakistan.

The Random Forest classification of coastal landforms along Pakistan’s coastline (2020–2024) delineated five primary LULC classes: mangroves, mudflats, water bodies, land parcels, and sand surfaces. Time-series analysis revealed a net expansion in mangrove extent from approximately 855 km^2^ in 2020 to 969 km^2^ in 2023, followed by a slight reduction to 947 km^2^ in 2024, with the peak estimate closely aligning with independent national assessments 996.28 km^2^. Mangrove forests were predominantly concentrated in the Indus Delta (Sindh province), forming dense, contiguous stands, while the Balochistan (Makran) coast exhibited fragmented distribution confined to sheltered embayments including Miani Hor, Kalmat Khor, and Gwadar Bay (Figure 4).

**Figure 5:**
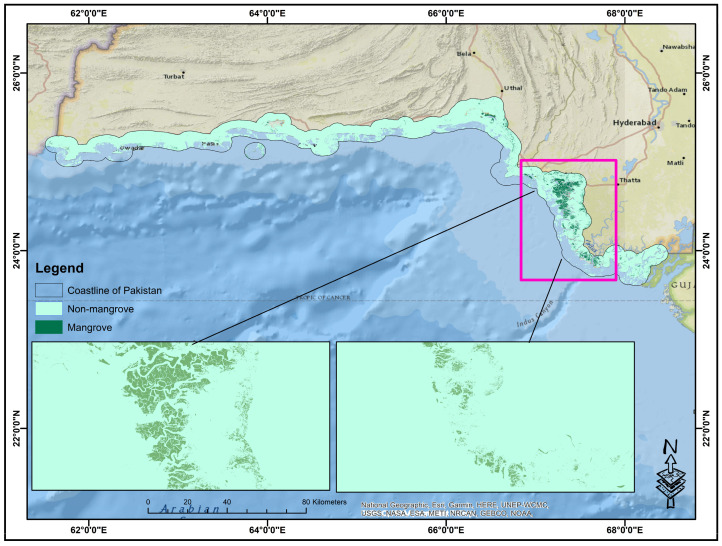
Spatial identification area of high mangrove density along the coastline of Pakistan.

Within the Indus Delta, the highest mangrove density and biomass were observed in the central region surrounding Keti Bunder and Kharo Chan, where riverine sediment supply and tidal flushing create favorable conditions for *Avicennia marina* growth. This area, spanning approximately 420 km^2^ consistently exhibited NDVI values exceeding 0.60 and carbon stocks above 40 Mg C ha^−1^ across all study years, representing the primary carbon stock “area of high mangrove density” along the Pakistan coast (Figure 5). In contrast, the eastern and western fringes of the delta showed lower mangrove density with NDVI values ranging from 0.35 to 0.50, corresponding to carbon stocks of 20–30 Mg C ha^−1^, primarily due to higher salinity intrusion and reduced freshwater influence.

**Figure 6:**
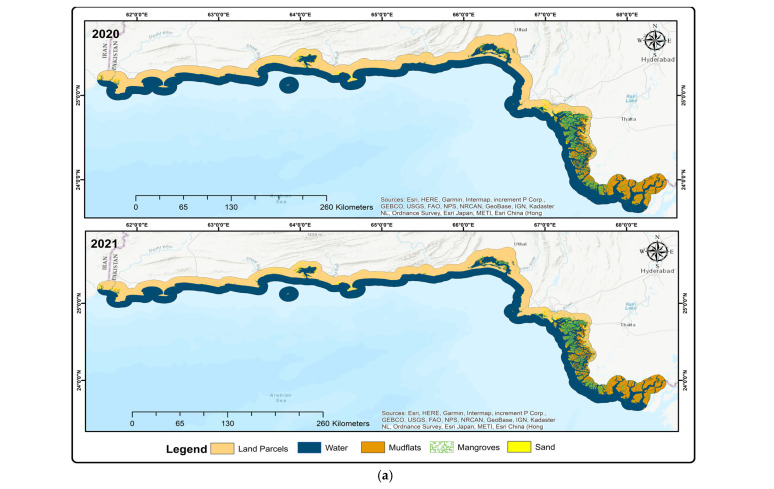

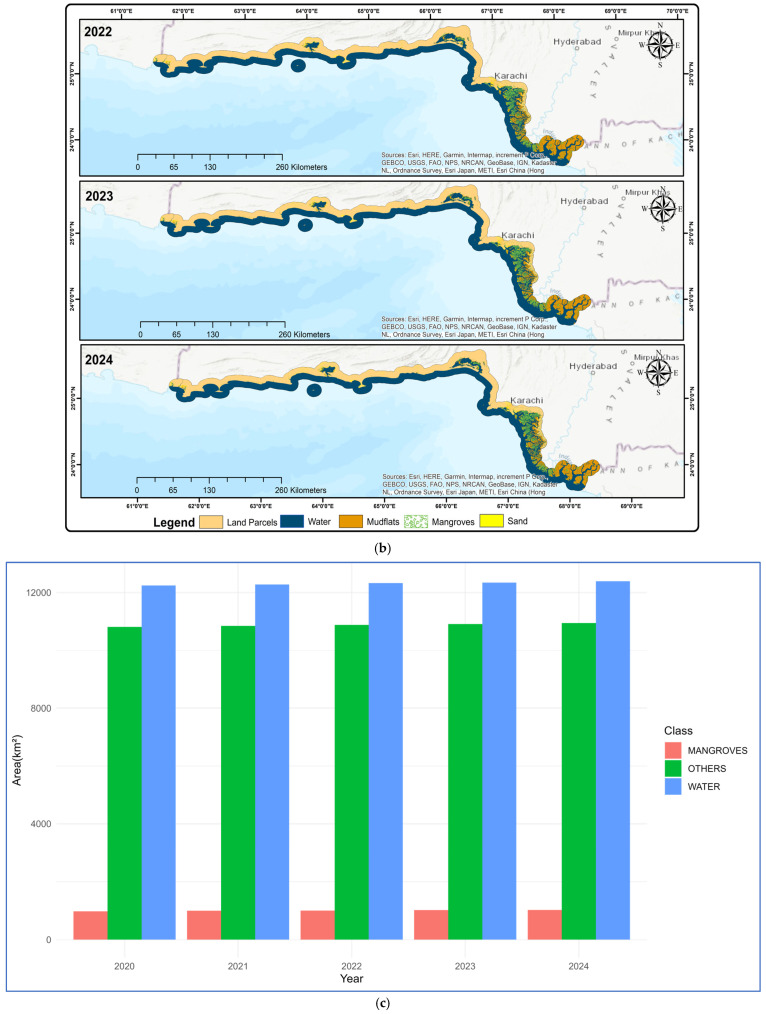
(**a**) Spatiotemporal evolution of coastal land use and land cover (LULC) for the year 2020 and 2021. (**b**) Spatiotemporal evolution of coastal land use and land cover (LULC) for the year 2022 to 2024. (**c**) Spatiotemporal variation in mangrove extent and other classes along the Pakistan coast from 2020 to 2024 derived from Landsat imagery.

Furthermore, Figure 6a,b revealed that the spatiotemporal LULC mapping from 2020 to 2024 shows that mangroves remained consistently concentrated in the eastern Indus Delta, while mudflats occupied the dynamic intertidal zone. Water bodies remained stable offshore, whereas land parcels and sand surfaces showed minimal interannual variation, serving as stable terrestrial baselines that validated classification consistency.

Figure 6c shows a net positive increase across the LULC matrix, where mangrove ecosystems expanded by approximately 5.1% over the 5-year observational window. This concurrent expansion of both the marine (Water’) and vegetative (Mangrove’) baselines reflect a highly dynamic shoreline advancement or a localized increase in tidal flooding patterns that support vegetation recruitment.

**Figure 7:**
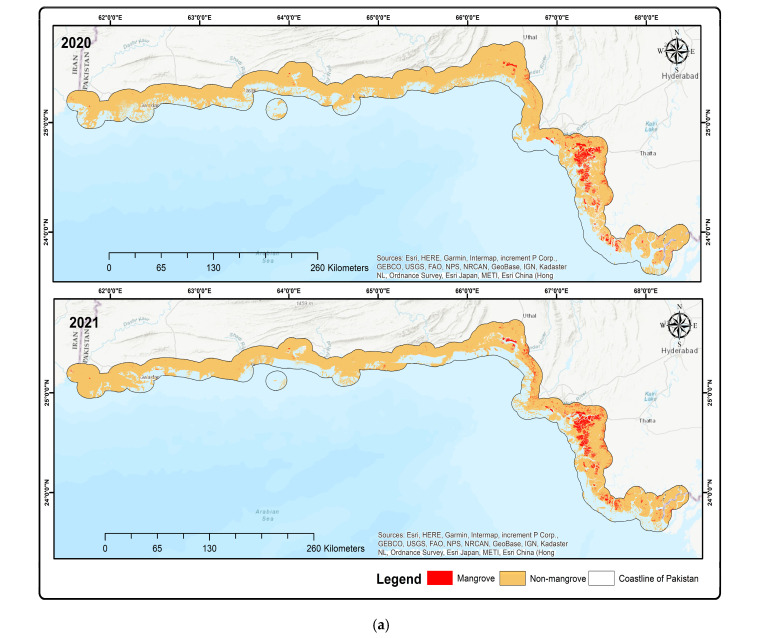

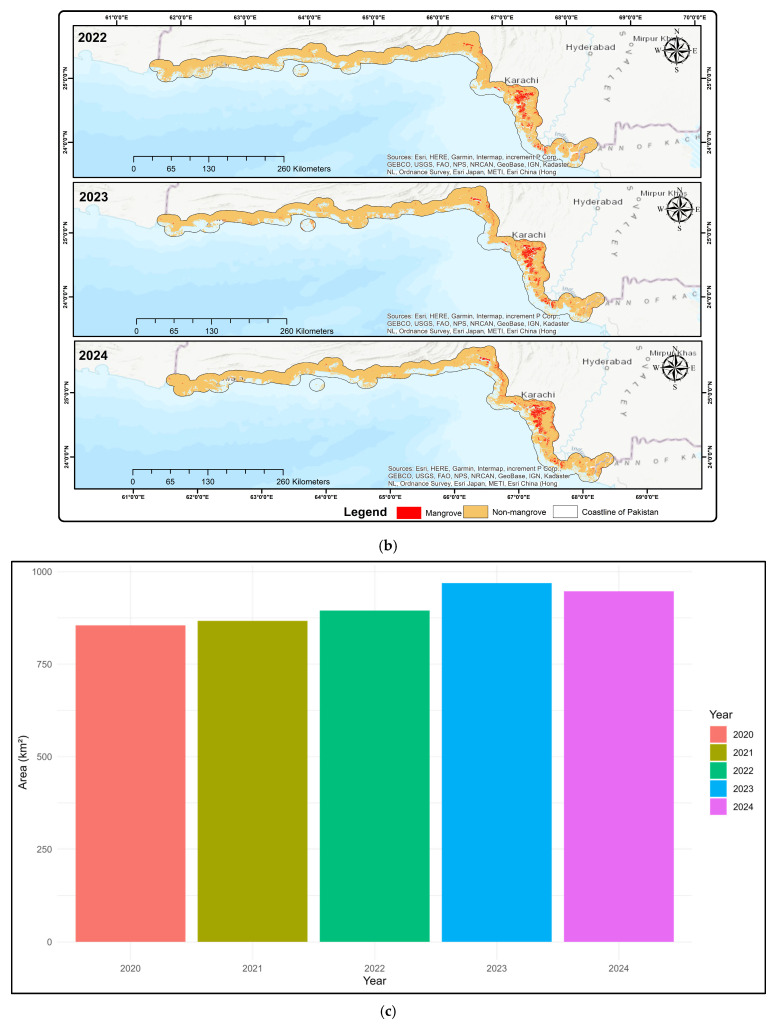
(**a**) Binary spatiotemporal distribution of mangrove forests (2020–2021). (**b**) Binary spatiotemporal distribution of mangrove forests from 2022 to 2024. (**c**) Spatiotemporal variation in mangrove along the Pakistan coast from 2020 to 2024 derived from Sentinel-2 imagery.

Figure 7a–c revealed that the temporal analysis of mangrove surface extent from 2020 to 2024 shows a progressive annual expansion that peaks significantly in 2023, followed by a slight, minor contraction in 2024. This overall positive net growth across the five-year observational window highlights a resilient vegetative baseline, likely driven by localized restoration success or favorable intertidal hydrological shifts before stabilizing. The spatial analysis was performed using Sentinel-2 satellite imagery, leveraging its high spatial resolution (up to 10 m) to ensure precise delineation of coastal boundaries and accurate mapping of localized mangrove distribution trends.

### 3.2. NDVI-Based Vegetation Dynamics and Canopy Vigor

Interannual analysis of the NDVI indicated persistent healthy mangrove canopies along the entire coastline of Pakistan from 2020 to 2024, as illustrated in Table 2. The table shows that the mean NDVI values were 0.58 in 2020, 0.55 in 2021, 0.58 in 2022, 0.56 in 2023, and 0.57 in 2024. The lowest NDVI (0.55) occurred in 2021, corresponding to a 11.11% decline in carbon stocks (3.56 Mg C ha^−1^) and coinciding with the highest vapor pressure deficit (VPD = 1.426 kPa) recorded during the study period. Recovery to 0.58 in 2022 and sustained values above 0.55 across all remaining years confirmed ecological stability and the absence of systemic degradation. These NDVI trajectories, characterized by oscillatory rather than monotonic patterns, suggest that mangrove canopies in this arid environment exist in a state of dynamic equilibrium governed primarily by interannual moisture availability rather than progressive structural decline.

### 3.3. Classification Accuracy Assessment

Classification accuracy was evaluated using 57 field-verified ground truth points distributed along the Sindh coastline. The spatial distribution of these 57 ground validation points is shown in Figure 3. Sentinel-2 (10 m resolution) consistently outperformed Landsat (30 m) Random Forest classification (Table 3). Sentinel-2 achieved overall accuracy (OA) ranging from 89.5% (2021) to 100% (2024), with perfect user accuracy (1.000) for mangrove class in all years. Landsat OA ranged from 88% (2021) to 99% (2024), with Kappa coefficients from 0.845 (2021) to 0.984 (2024). Sentinel-2 Kappa reached 1.000 in 2024. These results establish Sentinel-2 as the high-precision baseline for species-specific mangrove monitoring and carbon modeling.

### 3.4. Environmental Drivers and Light Use Efficiency

**Figure 8:**
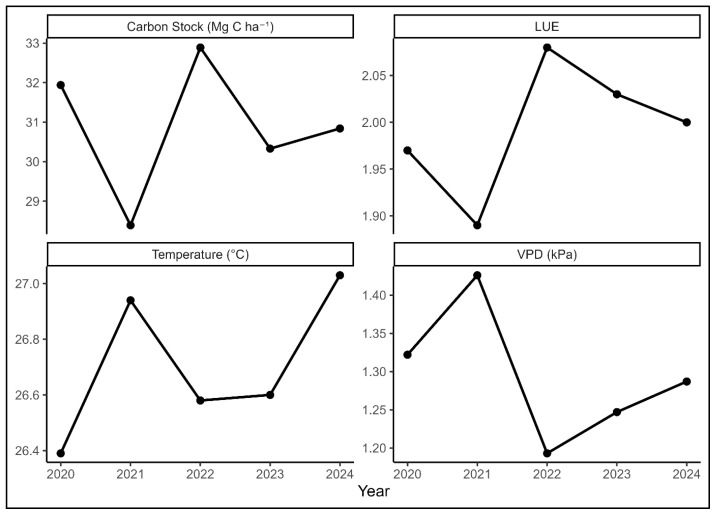
Multi-panel time series of carbon stock (Mg C ha^−1^), light use efficiency (LUE), temperature (°C), and vapor pressure deficit (VPD) from 2020 to 2024.

Interannual light use efficiency (LUE) analysis revealed a strong inverse relationship with vapor pressure deficit (VPD), as illustrated in Table 4. Minimum LUE (1.89) occurred in 2021 under peak water stress (water scalar $W_{s}$ = 0.76) and maximum VPD (1.426 kPa). Optimal moisture availability ($W_{s}$ = 0.83) and minimal VPD (1.193 kPa) in 2022 coincided with maximum LUE (2.08). The multi-panel time series in Figure 8 illustrates the interannual dynamics of carbon stock, LUE, temperature, and VPD from 2020 to 2024.

**Figure 9:**
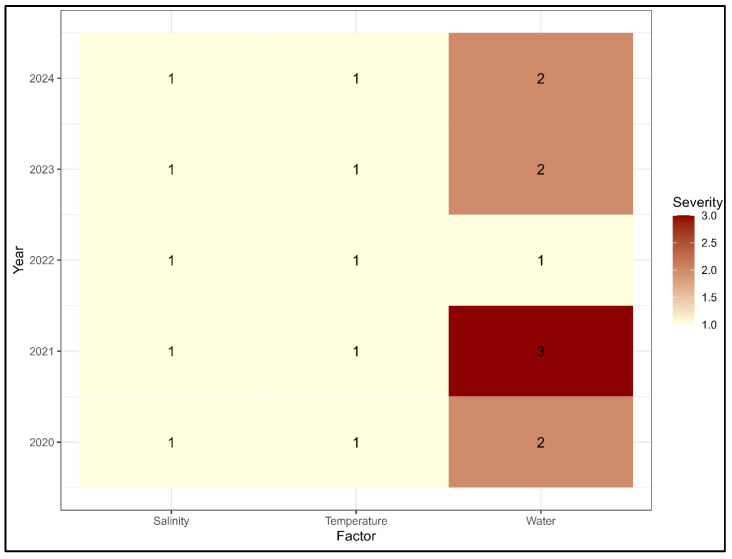
Heatmap of environmental stressor severity (2020–2024), showing water availability as the primary limiting factor.

Water availability was the dominant limiting factor in four of five years, with $W_{s}$ values ranging from 0.76 to 0.83. Temperature stress remained non-limiting throughout ($T_{s}=0.96–0.98$), with mean annual temperatures varying narrowly between 26.39 °C and 27.03 °C. The relative severity of each environmental stressor is visualized in the heatmap (Figure 9), which confirms water availability as the principal limiting factor across all years. The heatmap shows drought severity peaking in 2021 (darkest red) and minimal stress occurring in 2022 (lighter shades), consistent with the LUE and VPD trends reported above.

Salinity imposed a secondary, static constraint ($S_{s}=0.83$ invariant across all years). In 2022, transient moisture relief elevated $W_{s}$ to 0.83, equaling the salinity scalar and inducing definitive co-limitation (water + salinity). The radiation scalar ($P_{s}$) was fixed at 0.90 across all intervals.

### 3.5. Biomass and Carbon Stock Dynamics

**Figure 10:**
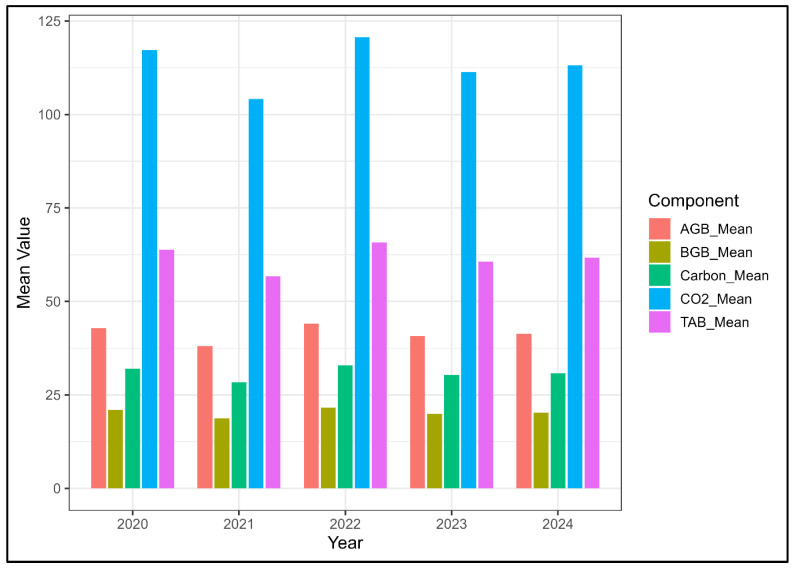
Annual mean dynamics of biomass (AGB, BGB, and TAB), carbon stocks, and CO_2_ equivalents.

Mean carbon stock across the entire study area (2020–2024) was 31.95 Mg C ha^−1^ (equivalent to 117.3 Mg CO_2_ ha^−1^), with significant interannual variation (coefficient of variation = 19.8%). Aboveground biomass (AGB) ranged from 38.10 to 44.14 Mg ha^−1^, and total accumulated biomass (TAB) from 56.77 to 65.77 Mg ha^−1^. Figure 10 displays the annual mean dynamics of biomass components (AGB, BGB, and TAB), carbon stocks, and CO_2_ equivalents from 2020 to 2024.

The complete yearly distribution of biomass components and carbon stocks, including ranges and means, is presented in Table 5. Annual carbon stock values were 31.94 Mg C ha^−1^ in 2020, declined to 28.38 Mg C ha^−1^ in 2021 (11.1% relative to 2020), peaked at 32.88 Mg C ha^−1^ in 2022, and then stabilized at 30.32 Mg C ha^−1^ in 2023 and 30.83 Mg C ha^−1^ in 2024.

Spatial heterogeneity in carbon distribution ranged from 21.25 to 55.94 Mg C ha^−1^ (min–max across years), primarily influenced by localized salinity gradients and water stress conditions. The annual statistical distribution of carbon stocks, including minimum, maximum, standard deviation, and coefficient of variation, is summarized in Table 6.

**Figure 11:**
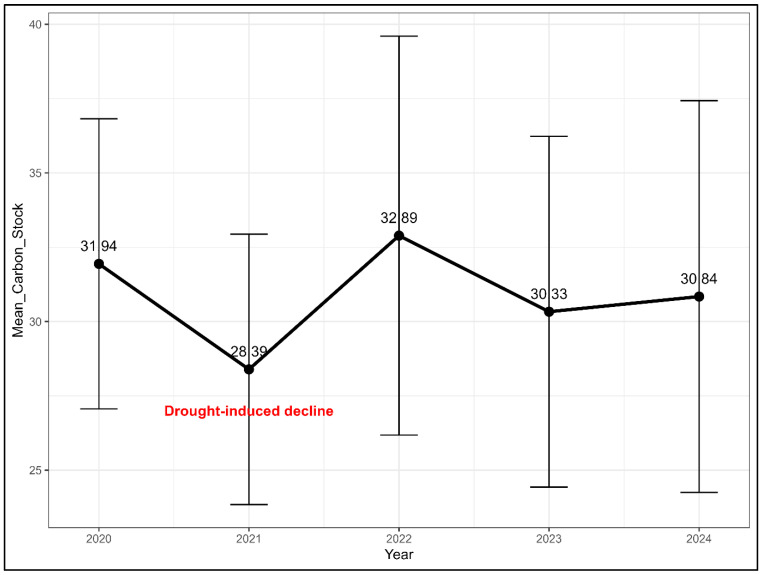
Mean carbon stock fluctuations and associated variance across the five-year study period.

The coefficient of variation (CV) for carbon stocks increased progressively from 15.3% (2020) to 21.4% (2024), indicating intensifying spatiotemporal heterogeneity. Figure 11 shows carbon stock fluctuations and associated variance across the five-year study period. The root-to-shoot ratio (belowground biomass = 0.49 × AGB) remained stable, confirming consistent allometric partitioning.

### 3.6. Ground Validation at Fixed Points

**Figure 12:**
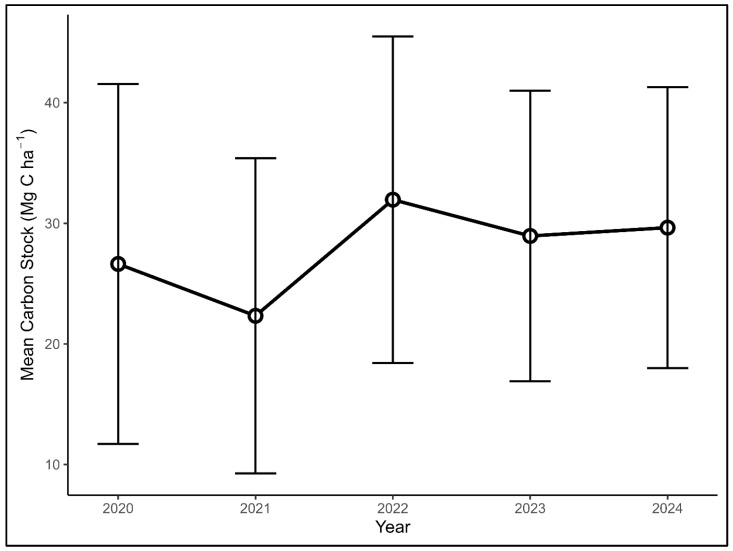
Annual mean carbon stock fluctuations and associated spatial variance.

Modeled biomass values were extracted at 57 georeferenced field points distributed across the coastline of Pakistan. Of these, 51 points supported active mangrove vegetation, while 6 non-vegetated points served as baseline references for calibration. Mean carbon stock at the 51 vegetated points was 27.90 ± 13.04 Mg C ha^−1^, with a coefficient of variation (CV) of 46.7% (Table 7). Annual point-specific carbon stocks exhibited marked interannual variability. Mean carbon stock declined from 26.63 ± 14.93 Mg C ha^−1^ in 2020 to 22.33 ± 13.06 Mg C ha^−1^ in 2021, representing a 16% reduction coincident with the peak VPD (1.426 kPa) and minimum LUE (1.89) recorded during the study period. In 2022, carbon stock recovered to 31.96 ± 13.54 Mg C ha^−1^, exceeding pre-drought levels and corresponding to the lowest VPD (1.193 kPa) and maximum LUE (2.08). Subsequently, carbon stock stabilized at 28.95 ± 12.05 Mg C ha^−1^ in 2023 and 29.64 ± 11.64 Mg C ha^−1^ in 2024 (Figure 12).

The CV at the point scale ranged from 39.3% (2024) to 58.5% (2021), substantially higher than the regional CV (15.3–21.4%). This disparity reflects the true structural heterogeneity of the landscape, which comprises a mosaic of mature forest stands, active restoration sites, and fragmented mudflat margins that are smoothed when aggregated to regional averages.

The rapid recovery from 22.33 Mg C ha^−1^ (2021) to 31.96 Mg C ha^−1^ (2022) within a single year demonstrates high phenotypic plasticity and ecosystem resilience in *Avicennia marina*-dominated mangroves under pulsed hydrological relief. This post-drought rebound confirms that short-term carbon stock declines in this arid system are driven by physiological suppression rather than structural deforestation.

### 3.7. Regression and Correlation Analyses

**Figure 13:**
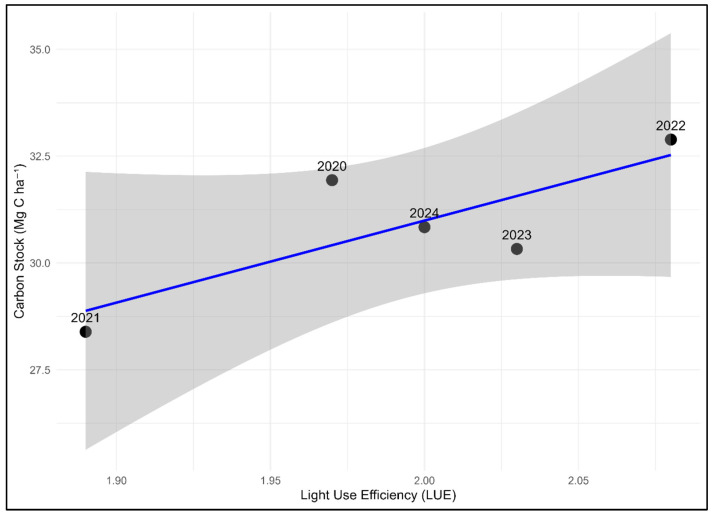
Regression of carbon stock against light use efficiency.

To evaluate the predictive strength of light use efficiency (LUE) and vapor pressure deficit (VPD) on mangrove carbon dynamics, linear regression analyses were conducted between carbon stock and each environmental variable. Carbon stock exhibited a significant positive linear relationship with LUE (R^2^ = 0.89, *p* < 0.05), confirming that LUE is a robust predictor of mangrove productivity in this arid coastal system. As shown in Figure 13, higher LUE values correspond systematically to greater carbon stocks, with the 2022 maximum LUE (2.08) coinciding with the peak carbon stock (32.88 Mg C ha^−1^) and the 2021 minimum LUE (1.89) corresponding to the lowest carbon stock (28.39 Mg C ha^−1^). Conversely, an inverse relationship was observed between carbon stock and VPD (R^2^ = 0.76, *p* < 0.05). The 2021 drought conditions, characterized by the highest VPD (1.426 kPa), coincided with the minimum carbon stock (28.39 Mg C ha^−1^), while the 2022 minimum VPD (1.193 kPa) aligned with peak carbon sequestration (32.88 Mg C ha^−1^).

**Figure 14:**
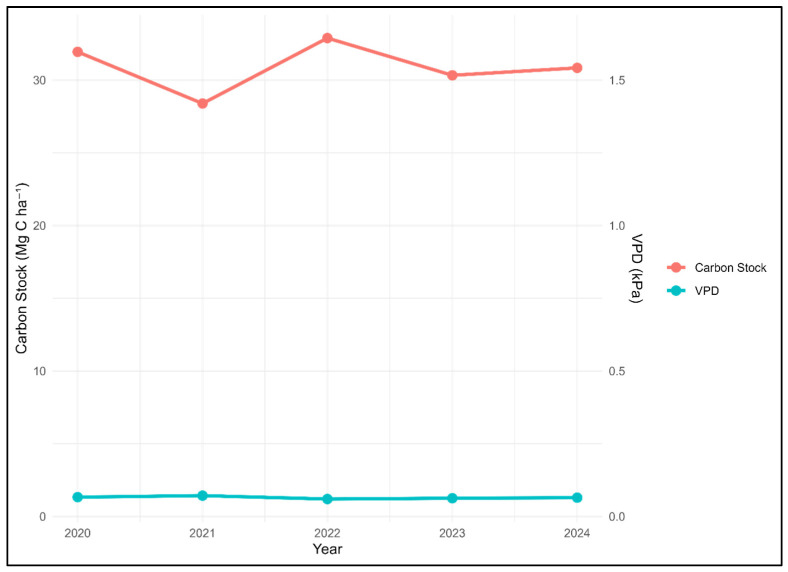
Annual trends in carbon stock relative to vapor pressure deficit (VPD) dynamics.

This inverse relationship is illustrated in Figure 14, which shows annual trends in carbon stock relative to VPD dynamics. These results indicate that atmospheric moisture demand, rather than temperature variation, is the primary meteorological control on interannual carbon sequestration in Pakistan’s arid-zone mangroves.

## 4. Discussion

### 4.1. Biomass and Carbon Stocks in Arid-Zone Mangroves

The observed aboveground biomass (AGB) range of 38.10–44.14 Mg ha^−1^ and total accumulated biomass (TAB) of 56.77–65.77 Mg ha^−1^ characterize Pakistan’s mangrove systems as moderate- to low-productivity carbon sinks when compared to humid tropical counterparts. This divergence is primarily driven by the hyper-arid and semi-arid conditions of the Indus Delta and Balochistan, where the dominance of *Avicennia marina* reflects specialized adaptation to hyper salinity and high vapor pressure deficit (VPD). Unlike the multi-species, high-stature forests of the Sundarbans or Southeast Asian estuaries, where AGB often exceeds 150–250 Mg ha^−1^ due to consistent precipitation and lower salinity [33,63,64], the Pakistani stands exhibit structural dwarfism as a survival mechanism.

These findings are further put into perspective by contrasting them with mangrove ecosystems worldwide: Mangrove carbon reserves in Pakistan are within a moderate global range, according to Meng et al. [65]. In China, AGB values range from 12.0 to 150.2 Mg ha^−1^, whereas BGB values range from 46.6 to 388.6 Mg ha^−1^. Hickey et al. [66] reported 70 Mg ha^−1^ in Australia, whereas Pham et al. [67] found mean AGB in Vietnam ranging from 51.58 to 79.90 Mg ha^−1^. These findings are comparable to the current findings. According to this comparison, the mangroves on Pakistan’s coastline continue to have biomass levels that are marginally lower than those of other tropical and subtropical mangrove systems worldwide. This is probably due to the arid to semi-arid conditions of the Indus Delta environment, but they are still important regional carbon sinks. The study’s AGB:BGB ratio (about 2:1) is in line with global trends in deltaic and semi-arid mangrove forests, where belowground allocation fosters sediment stability and resistance against hydrological stress [65,68]. In arid mangrove habitats such as the Indus River Delta, BGB often constitutes a significant amount of total biomass due to environmental limitations that limit shoot growth while maintaining robust root systems.

A root-to-shoot ratio (BGB: AGB) of 0.49 was used for this inquiry based on information from regional and local investigations. *Avicennia marina* in the Indus Delta exhibits little aboveground growth when exposed to high saline conditions (28–32 PSU), while root activity remains strong, leading to a naturally improved root-to-shoot allocation [36]. The same species under similar saline and arid conditions showed BGB equivalent to about 80% of AGB, according to research [64]. Komiyama et al. [63] further supported the phenomenon of significant belowground investment in stressed mangrove systems by demonstrating that BGB can match or exceed AGB in temperate mangroves. In addition to AGB = 112 t ha^−1^ and BGB = 160 t ha^−1^, they also reported occurrences of AGB = 144 t ha^−1^ and BGB = 147 t ha^−1^. Because the Indus Delta shares environmental constraints with these documented occurrences, particularly about aridity and salinity stress, the low value of 0.49 is a wise and environmentally acceptable choice for biomass estimation. Using this ratio ensures that BGB contributions are accurately reflected in carbon stock calculations because belowground components are essential to carbon sequestration in arid mangrove forests. This method is consistent with the criteria for wetland biomass and provides a strong basis for estimating total mangrove biomass in regions where environmental stress limits aboveground growth while promoting substantial root development.

Our findings of a ~2:1 AGB:BGB ratio (root-to-shoot ratio of 0.49) align with resource allocation theory, suggesting that plants under hydrological stress prioritize belowground investment to enhance nutrient uptake and mechanical stability in unstable, saline sediments [14,61]. This conservative allocation ensures accurate carbon quantification in the Indus Delta, where environmental constraints parallel other arid-saline systems globally, such as those in Oman and western Australia [64,65].

### 4.2. Decoupling of Temperature and Productivity

A critical finding of this study is the decoupling of thermal regimes from productivity. While temperature stress scalars remained near-optimal (0.96–0.98), interannual fluctuations in light use efficiency (LUE) and biomass were strictly dictated by atmospheric moisture demand. The 2021 drought paradox where peak VPD (1.426 kPa) induced significant carbon uptake suppression despite optimal temperatures suggests that stomatal conductance is restricted to prevent xylem cavitation, a hallmark of arid-zone mangrove physiology [59,60].

### 4.3. The 2022 Anomaly: Pulse Resilience

Conversely, the 2022 productivity peak (AGB: 44.14 Mg ha^−1^) marks a significant ecological anomaly driven by extreme monsoon and subsequent flooding that temporarily redefined the region’s environmental constraints. While the 2022 floods were catastrophic for infrastructure, they acted as a high-magnitude resource pulse for mangroves in both Sindh and Balochistan. The primary driver was the reduction in VPD to a five-year minimum of 1.193 kPa. In this hyper-arid belt, mangroves typically experience atmospheric “thirst” that forces stomatal closure to prevent water loss; however, the saturated air of 2022 allowed *Avicennia marina* to maintain stomatal conductance for longer periods, maximizing photosynthetic efficiency and pushing biomass to its study-period maximum [68,69].

Furthermore, massive freshwater discharge from the Indus River and flash floods from the Kirthar and Suleman ranges transformed coastal soil chemistry through salinity alleviation, flushing out hypersaline pore-water and moving the system toward a more physiologically optimal state [36]. These floodwaters introduced significant nutrient loading and terrestrial sediment, which fueled canopy expansion and provided fresh substrate for natural recruitment. In Balochistan, rare flash floods through ephemeral streams provided a similar boost to otherwise fragmented stands. Collectively, these factors created a window where the usual limits of aridity were lifted, demonstrating that Pakistan’s coastal forests possess a remarkable capacity for compensatory greening. The 2022 data confirms that these ecosystems are pulse-resilient, where gains made during extreme wet years can offset periods of moderate drought stress.

### 4.4. Spatial Heterogeneity and Scaling Implications

The discrepancy between regional variability (CV: 15.3–21.4%) and point-scale validation (CV: 39.3–58.5%) further highlights the importance of spatial aggregation in these dynamic systems. While regional integration smooths micro-scale gradients for broad-scale monitoring, the substantially higher standard deviation at georeferenced points captures the true structural heterogeneity of the landscape, a mosaic of mature forests, recent restoration sites, and fragmented mudflats. This finding aligns with previous studies in semi-arid mangroves, which emphasize that aggregated estimates may mask critical fine-scale variability [37,67,70].

The rapid recovery observed in 2022 following the 2021 decline illustrates a high degree of drought-response plasticity in *Avicennia marina*. The ecosystem exists in a state of dynamic equilibrium, where biomass trajectories reflect genuine eco-physiological responses to moisture availability rather than systematic degradation. While Pakistan’s carbon stocks remain lower than humid tropical systems (e.g., Vietnam or China), they constitute vital regional sinks for blue carbon.

### 4.5. Methodological Validation and Broader Implications

The alignment of our extent estimates (~969 km^2^) with independent national assessments at 996.28 km^2^ affirms the validity of this nested remote sensing approach [61]. By combining regional climatic driver identification with ground-anchored validation, this research offers a robust framework for carbon accounting that recognizes the specific eco-physiological triggers of the Pakistani coastline rather than relying on generalized tropical models. This approach is directly applicable to REDD+ initiatives and national carbon inventory frameworks in arid and semi-arid coastal zones globally.

## 5. Conclusions

This study quantified mangrove carbon stock dynamics along Pakistan’s coastline (2020–2024) using a light use efficiency model. Mangrove extent expanded from 855 km^2^ to 969 km^2^ (2023), and then slightly reduced to 947 km^2^ (2024), aligning with independent assessments (996.28 km^2^. Mean carbon stock was 31.95 Mg C ha^−1^ (CV = 19.8%). Water availability (VPD) was the dominant limiting factor, while temperature remained non-limiting (26.39–27.03 °C). The 2021 drought (VPD = 1.426 kPa) caused a 11.11% carbon decline, followed by rapid 2022 recovery (VPD = 1.193 kPa), demonstrating pulse resilience in *Avicennia marina*. Spatial heterogeneity was substantial (regional CV: 15.3–21.4%; point-scale CV: 39.3–58.5%), emphasizing the need for fine-scale validation.

This study makes several methodological and scientific contributions to blue carbon assessment in arid-zone mangroves. First, it demonstrates the operational applicability of a process-based LUE model in data-scarce, hyper-arid coastal environments where field measurements are logistically challenging. Second, by integrating 57 ground-validation points with multi-source satellite data (Sentinel-2 at 10 m resolution and Landsat 8–9), the nested approach bridges the gap between regional-scale carbon accounting and point-scale ecological heterogeneity. Third, this study provides the first high-resolution (10 m), multi-year (2020–2024) quantification of mangrove carbon stocks along the full Pakistan coastline, identifying VPD as the primary meteorological control rather than temperature, a finding that challenges generalized tropical carbon models. Fourth, the 2021–2022 drought–flood pulse analysis offers empirical evidence of phenotypic plasticity and ecosystem resilience in *Avicennia marina*, contributing to understanding of mangrove responses to climate extremes. Finally, the validated extent estimates (~969 km^2^) and carbon stock baselines establish a robust reference for national REDD+ reporting and IPCC greenhouse gas inventories. This study demonstrates a replicable, cost-neutral workflow for routine blue carbon monitoring, offering an evidence base for protection and restoration of Pakistan’s coastal mangrove ecosystems.

## Figures and Tables

**Table 1:** Overview of dataset specifications and classification accuracy metrics for coastal monitoring (2020–2024).

Parameter	Description
Number of ground points	57
Spatial coverage	Sindh and Balochistan coastline
Classification	Mangrove (1), non-mangrove (0)
Validation years	2020, 2021, 2022, 2023, 2024
Satellite data	Sentinel-2, 10 m resolution
Accuracy metrics	Overall, Producer’s, User’s, Kappa

**Table 2:** Annual observed mean NDVI value interpretation for the years of 2020–2024.

Year	OM NDVI ^1^	Interpretation
2020	0.58	Peak vegetation health; dense and vigorous mangrove canopy.
2021	0.55	Slight decline in greenness; possible minor stress or seasonal variation.
2022	0.58	Recovery to peak levels; sustained density and high chlorophyll activity.
2023	0.56	Minor fluctuation; stable health with slight decrease in canopy density.
2024	0.57	Stable vegetation health; maintaining a healthy and robust mangrove cover.

^1^ Observed Mean NDVI.

**Table 3:** Comparative accuracy assessment of Landsat (RF) and Sentinel-2 for mangrove classification (2020–2024).

Year	Data Source	Overall Accuracy (%)	Producer’s Accuracy	User’s Accuracy	Kappa Coefficient
2020	A	94.0	0.920	0.930	0.912
	B	96.5	0.965	1.000	0.930
2021	A	88.0	0.880	0.890	0.845
	B	89.5	0.895	1.000	0.790
2022	A	89.0	0.860	0.870	0.810
	B	91.2	0.912	1.000	0.820
2023	A	92.0	0.900	0.910	0.864
	B	96.5	0.965	1.000	0.930
2024	A	99.0	0.800	1.000	0.984
	B	100.0	1.000	1.000	1.000

A represents Landsat (RF), while B represents Sentinel-2.

**Table 4:** Annual environmental variables, light use efficiency (LUE), and primary limiting factors for mangrove growth (2020–2024).

Year	T (°C)	$T_{dew}$ (°C)	VPD (kPa)	$T_{s}$	c	$S_{s}$	$P_{s}$	LUE	Limiting Factor
2020	26.39	18.41	1.322	0.96	0.79	0.83	0.90	1.97	Water
2021	26.94	18.48	1.426	0.98	0.76	0.83	0.90	1.89	Water (drought)
2022	26.58	19.68	1.193	0.96	0.83	0.83	0.90	2.08	Water + Salinity
2023	26.60	19.28	1.247	0.97	0.81	0.83	0.90	2.03	Water
2024	27.03	19.67	1.287	0.98	0.80	0.83	0.90	2.00	Water

**Table 5:** Yearly biomass and carbon stock distribution across the coastline of Pakistan.

Year	2020	2021	2022	2023	2024
AGB Range	29.07–72.56	27.51–68.75	30.03–75.08	28.74–71.87	28.52–71.32
AGB Mean	42.87	38.1	44.14	40.7	41.39
BGB Range	14.24–35.56	13.48–33.69	14.71–36.79	14.08–35.21	13.97–34.94
BGB Mean	21.01	18.67	21.63	19.94	20.28
TAB Range	43.32–108.12	41.00–102.44	44.75–111.87	42.83–107.09	42.50–106.28
TAB Mean	63.88	56.77	65.77	60.65	61.67
Carbon range	21.66–54.06	20.51–51.22	22.37–55.93	21.41–53.57	21.25–53.13
Carbon mean	31.94	28.38	32.88	30.32	30.83
CO2 range	79.50–198.40	75.23–187.99	82.11–205.29	78.60–196.50	78–195
CO2 mean	117.23	104.18	120.63	111.3	113.17

**Table 6:** Annual variation in carbon stock distribution and statistical dispersion metrics.

Year	MIN (Mg C ha^−1^)	MAX (Mg C ha^−1^)	MEAN (Mg C ha^−1^)	STD (Mg C ha^−1^)	CV (%)
2020	21.66	54.06	31.94	4.88	15.3
2021	20.51	51.22	28.39	4.55	16.0
2022	22.38	55.94	32.89	6.71	20.4
2023	21.42	53.54	30.33	5.90	19.5
2024	21.25	53.13	30.84	6.59	21.4

**Table 7:** Summary of annual mean carbon stock density and statistical dispersion.

Year	Mean Carbon Stock (Mg C ha^−1^)	SD Carbon	CV (%)
2020	26.63	14.93	56.1
2021	22.33	13.06	58.5
2022	31.96	13.54	42.4
2023	28.95	12.05	41.6
2024	29.64	11.64	39.3
Overall (2020–2024)	27.90	13.04	46.7

## Data Availability

The data presented in this study are available from the corresponding author on request.
